# Tissue-Resident Macrophages in Cardiovascular Diseases: Heterogeneity and Therapeutic Potential

**DOI:** 10.3390/ijms26104524

**Published:** 2025-05-09

**Authors:** Tianhui An, Mengyuan Guo, Zhaohui Wang, Kun Liu

**Affiliations:** 1 Department of Geriatrics, Union Hospital, Tongji Medical College, Huazhong University of Science and Technology, Wuhan 430022, China; zhaohuiwang@hust.edu.cn; 2 Department of Cardiology, Union Hospital, Tongji Medical College, Huazhong University of Science and Technology, Wuhan 430022, China; 2020xh5037@hust.edu.cn

**Keywords:** cardiovascular disease, tissue-resident macrophages, inflammation, metabolic reprogramming

## Abstract

Tissue-resident macrophages (TRMs) play a crucial role in maintaining tissue homeostasis and regulating immune responses. In recent years, an increasing number of studies have highlighted their central role in cardiovascular diseases. This review provides a comprehensive overview of TRMs, with a particular emphasis on cardiac resident macrophages (CRMs), discussing their origin, heterogeneity, and functions in various cardiovascular diseases. We conduct an in-depth analysis of macrophage subpopulations based on C-C Chemokine Receptor Type 2 (CCR2) receptor expression, elucidating the role of CCR2^+^ macrophages in promoting fibrosis and cardiac remodeling, while highlighting the protective functions of CCR2^−^ macrophages in suppressing inflammation and promoting tissue repair. In atherosclerosis, we focus on the role of metabolic reprogramming in regulating macrophage polarization, revealing how metabolic pathways influence the balance between pro-inflammatory M1 and anti-inflammatory M2 macrophages, thereby affecting plaque stability and disease progression. By summarizing the roles of these macrophage subpopulations in myocardial infarction, heart failure, and other diseases, we propose potential therapeutic strategies aimed at modulating different macrophage subtypes. These include targeting the CCR2 signaling pathway to mitigate inflammation and fibrosis, and metabolic reprogramming to restore the balance between M1 and M2 macrophages. Finally, we highlight the need for future research to focus on the functional diversity and molecular mechanisms of human TRMs to develop novel immunotherapeutic strategies and improve the prognosis of cardiovascular diseases.

## 1. Background

Macrophages, as a key component of the innate immune system, are widely distributed across various tissues, playing a central role in pathogen clearance, apoptotic cell phagocytosis, and immune regulation [1]. Recent research has increasingly highlighted the critical role of TRMs, which exhibit remarkable phenotypic heterogeneity and functional plasticity through epigenetic programming in response to microenvironmental cues, in not only maintaining tissue homeostasis but also dynamically balancing their roles as key modulators in the initiation and progression of diverse diseases [2]. In the cardiovascular system, TRMs play crucial roles in regulating pathological inflammation, fibrosis, and tissue repair, making them key mediators in the pathogenesis and progression of cardiovascular diseases [3].

**Figure 1:**
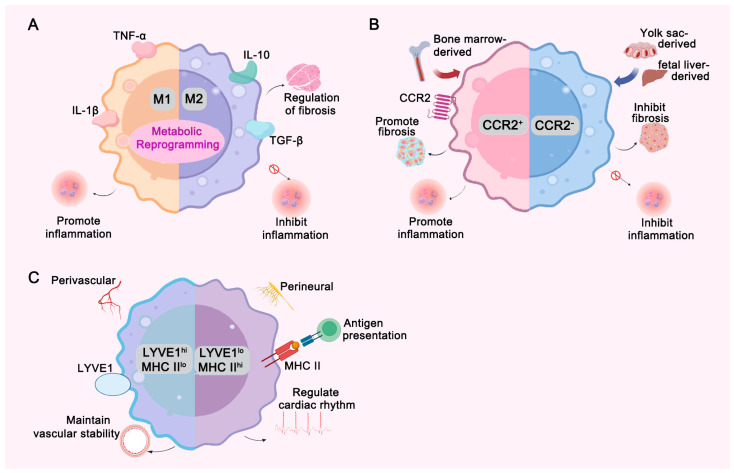
Phenotyping and Functions of Resident Macrophages. (**A**) Resident macrophages are divided into M1 and M2 subtypes. M1 macrophages secrete pro-inflammatory cytokines like TNF-α and IL-1β, promoting inflammation, while M2 macrophages secrete IL-10 and TGF-β, suppressing inflammation and moderately regulating fibrosis. Metabolic reprogramming plays a key role in the differentiation of M1 and M2. (**B**) Cardiac resident macrophages (CRMs) can be classified into CCR2^+^ and CCR2^−^ subtypes. CCR2^+^ macrophages, derived mainly from bone marrow monocytes, promote fibrosis and inflammation. CCR2^−^ macrophages, originating from the yolk sac and fetal liver, have anti-fibrotic and anti-inflammatory functions. (**C**) CRMs are further subdivided into LYVE1^hi MHC-II^lo and LYVE1^lo MHC-II^hi subsets. LYVE1^hi MHC-II^lo macrophages are located around blood vessels and may maintain vascular stability, while LYVE1^lo MHC-II^hi macrophages are found near nerve fibers and may be involved in neural regulation, antigen presentation, and heart rhythm control.

Traditionally, TRMs are categorized into two primary subtypes based on their activation states: M1 and M2 macrophages (Figure 1A). M1 macrophages, often termed “classically activated”, are predominantly involved in initiating early inflammatory responses and exerting antimicrobial effects through the secretion of pro-inflammatory cytokines, such as tumor necrosis factor-alpha (TNF-α) and interleukin-1β (IL-1β) [4,5]. Conversely, M2 macrophages, also known as “alternatively activated”, promote tissue repair and modulate fibrotic processes by releasing anti-inflammatory mediators such as interleukin-10 (IL-10) and transforming growth factor-beta (TGF-β), playing a crucial role in the resolution phase of inflammation and tissue repair [4,5]. The delicate balance between M1 and M2 macrophage populations is a key determinant in the pathogenesis and progression of cardiovascular diseases, particularly in conditions such as atherosclerosis.

Recent advances in the field of immunometabolism have revealed that metabolic reprogramming is a crucial regulator of macrophage polarization [6]. The alteration of metabolic pathways significantly affects the balance between pro-inflammatory M1 and anti-inflammatory M2 macrophages, thereby influencing plaque stability and the progression of disease [6,7]. This emerging understanding provides a novel therapeutic approach for atherosclerosis, as targeting immunometabolic pathways could offer new strategies to modulate macrophage function and potentially halt or reverse disease progression [7].

In cardiac tissue, CRMs exhibit not only M1/M2 functional heterogeneity but can also be divided into two subpopulations based on the expression of C-C chemokine receptor type 2 (CCR2): CCR2^−^ and CCR2^+^ macrophages (Figure 1B) [1]. CCR2^−^ macrophages primarily originate from embryonic hematopoietic sources, such as the yolk sac and fetal liver [8,9]. These cells migrate to the heart during early development, where they become resident and maintain their population through self-renewal, independent of circulating monocyte replenishment [10,11]. Widely distributed throughout various regions of the heart, CCR2^−^ macrophages are essential for coronary system development [12]. They play a critical role in mitigating excessive inflammatory responses and modulating fibrosis following myocardial infarction, thereby facilitating favorable cardiac remodeling [13]. In contrast, CCR2^+^ macrophages are predominantly derived from bone marrow monocytes in adulthood [12]. Under pathological cardiac conditions, such as myocardial infarction and heart failure, CCR2^+^ macrophages exhibit potent pro-inflammatory functions [12]. Characterized by high CCR2 expression, these cells are rapidly recruited to injured areas of the heart in response to chemokine signaling, where they initiate and sustain local inflammatory responses by secreting pro-inflammatory cytokines, including IL-1β, TNF-α, and IL-6 [14]. While this inflammatory response is critical for clearing necrotic tissue and limiting further damage during the early stages of myocardial infarction, excessive or prolonged inflammation can lead to adverse outcomes, such as exacerbated fibrosis and accelerated heart failure progression [15].

Chakarov and his colleagues further classified CRMs into two distinct subpopulations: LYVE1^hi MHC-II^lo and LYVE1^lo MHC-II^hi macrophages (Figure 1C) [16]. The LYVE1^hi MHC-II^lo subset, characterized by high expression of lymphatic vessel endothelial hyaluronan receptor 1 (LYVE1), is predominantly localized around the heart’s vasculature, particularly near the coronary arteries and capillary networks [16,17]. These macrophages are thought to contribute to vascular homeostasis by interacting with endothelial cells and facilitating vascular repair and regeneration through the release of vascular growth factors in response to injury [16,17]. In contrast, LYVE1^lo MHC-II^hi macrophages are predominantly localized near cardiac nerve fibers and are thought to be involved in neural regulation, antigen presentation, and the maintenance of normal cardiac rhythm, although their exact functions have yet to be fully elucidated [16,17]. Furthermore, CRMs may orchestrate cardiac repair processes by recruiting platelets to injury sites, where these circulating elements release platelet-derived growth factors (PDGFs) and serotonin. Such crosstalk potentially enables CRMs to fine-tune their functional specialization in resolving inflammation, mitigating fibrosis, and promoting tissue repair within the cardiac niche [18]. These findings highlight the functional heterogeneity of CRMs and suggest distinct roles for each subpopulation in cardiovascular homeostasis and disease.

This study aims to explore the heterogeneity of TRMs and their functions in cardiovascular diseases, particularly in mechanisms related to myocardial infarction, heart failure, atherosclerosis, hypertension, and pulmonary arterial hypertension. By analyzing the diversity of these macrophage subpopulations, we seek to identify potential therapeutic targets, specifically through interventions targeting the CCR2 signaling pathway and metabolic reprogramming to modulate the M1/M2 macrophage balance. These approaches may offer new strategies for the treatment of cardiovascular diseases in the future.

## 2. The Role of TRMs in Cardiovascular Disease

### 2.1. Myocardial Infarction

Myocardial infarction (MI) remains one of the leading causes of cardiovascular-related mortality worldwide, despite significant advancements in thrombolytic therapies and percutaneous coronary interventions [19]. The incidence of MI is notably high among elderly individuals and those with comorbid conditions such as hypertension and diabetes [19]. The primary pathological mechanism driving MI is ischemia-induced cardiomyocyte death, which triggers a cascade of complex pathological events [20]. Cardiomyocyte loss not only leads to localized functional impairment but also initiates a strong inflammatory response that exacerbates tissue damage [20,21]. This inflammatory reaction, combined with subsequent ventricular remodeling, greatly increases the risk of heart failure, further compromising the quality of life in affected patients [20,21].

**Figure 2:**
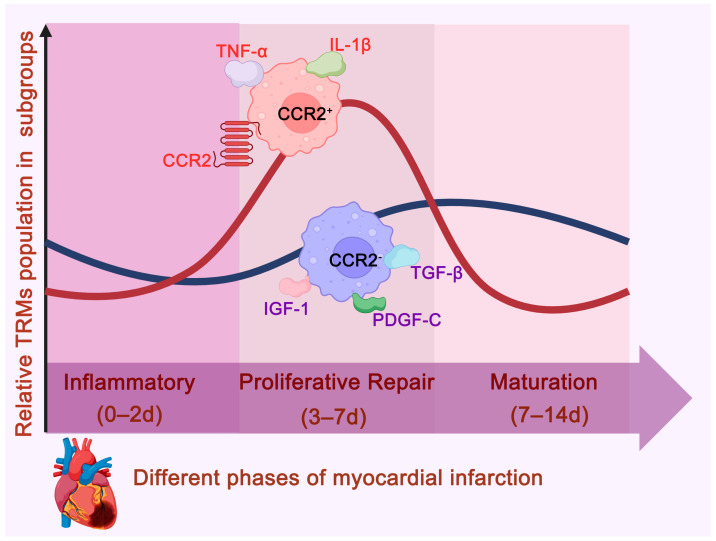
Dynamic Changes in Cardiac Resident Macrophages During Myocardial Infarction. The pathological progression of myocardial infarction (MI) can be divided into three phases: the inflammatory phase (0–2 days), the proliferative repair phase (3–7 days), and the maturation phase (7–14 days). Both CCR2^+^ and CCR2^−^ CRMs undergo a process of initial decline, followed by an increase, and then a subsequent decline. These CCR2^−^ macrophages play a protective role by reducing inflammation, preventing fibrosis, and promoting tissue repair through the secretion of TGF-β, IGF-1, and Platelet Derived Growth Factor C (PDGF-C). Conversely, CCR2^+^ macrophages contribute to chronic inflammation, fibrosis, and adverse cardiac remodeling by secreting TNF-α and IL-1β.

The pathological progression of myocardial infarction (MI) can be divided into three distinct but overlapping phases: the inflammatory phase, the proliferative repair phase, and the maturation phase (Figure 2) [22]. In the early inflammatory phase (0–2 days), the number of CRMs (including both CCR2^+^ and CCR2^−^ subtypes) decreases by approximately 50% in the infarcted region, while CRMs in distant areas undergo proliferative expansion [22]. CCR2^+^ macrophages, through the secretion of pro-inflammatory cytokines such as IL-1β and TNF-α, promote the recruitment of monocyte-derived macrophages to the infarct site, enhancing local inflammation and initiating tissue clearance [15,22,23]. In contrast to the prolonged activity of CCR2^+^ macrophages, which exacerbate inflammation, CCR2^−^ macrophages limit the spread of inflammation by secreting anti-inflammatory cytokines such as insulin-like growth factor 1 (IGF-1) and TGF-β [22].

During the proliferative repair phase (3–7 days), CCR2^+^ macrophages continue to proliferate, and their activity is closely linked to the progression of fibrosis and deterioration of cardiac function [22]. Research has shown that selective depletion of these pro-inflammatory cells can effectively mitigate the inflammatory response, reduce fibrosis, and significantly improve cardiac function [15,22,23]. CCR2^−^ macrophages, which gradually become dominant during the repair phase, secrete anti-inflammatory and reparative factors, such as IGF-1, TGF-β, and PDGF-C [15,22,23]. These factors inhibit the spread of inflammation, promote angiogenesis (essential for supplying oxygen and nutrients to metabolically active granulation tissue), and support myocardial tissue repair, thereby establishing the foundation for subsequent tissue remodeling [15,22,23]. Additionally, CCR2^−^ macrophages help clear apoptotic cardiomyocytes through a MerTK-dependent mechanism, reducing inflammation and accelerating tissue repair [15,22,24,25,26]. A lack of CCR2^−^ macrophages leads to poor infarct healing and further deterioration of cardiac function [22].

During the maturation phase (7–14 days), both CCR2^+^ and CCR2^−^ macrophages decrease, while cardiac fibroblasts migrate, proliferate, and produce collagen, leading to collagen deposition and mature scar formation [13,22]. As myocardial cell debris and macrophages are cleared via the cardiac lymphatic system, the inflammation gradually resolves [13,22,26].

From a therapeutic perspective, targeting CCR2^+^ macrophages represents a promising strategy to effectively reduce post-myocardial infarction inflammation, alleviate fibrosis, and improve cardiac function [23]. Studies have demonstrated that decreasing the recruitment and activity of CCR2^+^ macrophages can significantly lower the risk of fibrosis formation and reduce the likelihood of cardiac dysfunction [3]. Simultaneously, enhancing the reparative functions of CCR2^−^ macrophages may promote cardiac regeneration, inhibit fibrosis progression, and improve long-term outcomes for patients [3]. This opens new avenues for future research into MI interventions.

In summary, the distinct subtypes of CRMs play divergent roles in inflammation, fibrosis, and tissue repair following MI. CCR2^+^ macrophages primarily mediate pro-inflammatory and fibrotic responses, while CCR2^−^ macrophages actively suppress inflammation and promote tissue repair. By modulating the balance between these two macrophage populations, future therapeutic strategies may improve the long-term prognosis of MI patients and reduce the risk of end-stage heart conditions such as heart failure.

### 2.2. Heart Failure

**Figure 3:**
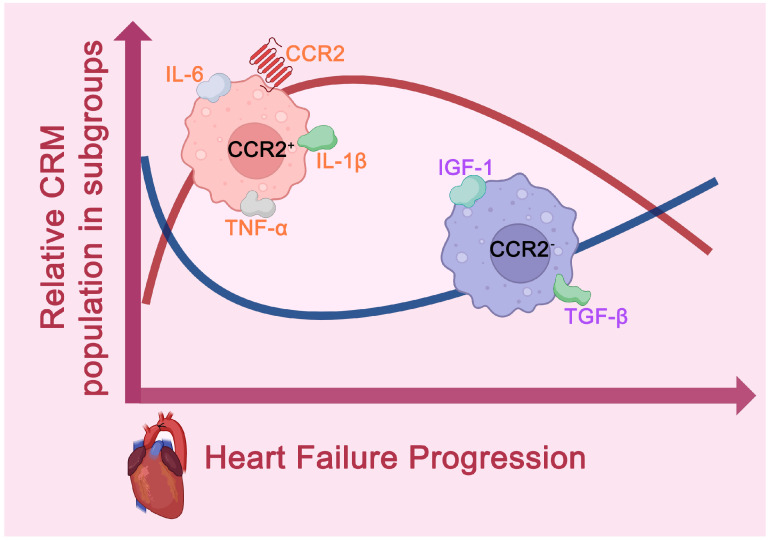
Dynamic Changes in Cardiac Resident Macrophages During Heart Failure. In the early stages of heart failure, CCR2^+^ macrophages quickly migrate to injury sites, releasing pro-inflammatory cytokines like IL-1β, TNF-α, and IL-6, which worsen inflammation, fibrosis, and cardiac remodeling. As the disease progresses, their numbers decrease. In contrast, CCR2^−^ macrophages provide protection early on but decline due to depletion, and they recover through proliferation in later stages. They contribute to anti-inflammatory and anti-fibrotic effects by secreting IGF-1 and TGF-β.

Heart failure (HF) is a cardiovascular disease with high global prevalence and mortality, characterized by a complex pathological process involving myocardial injury, inflammatory responses, fibrosis, and cardiac remodeling [27]. Based on the left ventricular ejection fraction (LVEF), HF can be classified into heart failure with reduced ejection fraction (HFrEF) and heart failure with preserved ejection fraction (HFpEF) [28]. In both subtypes, cardiac resident CCR2^+^ and CCR2^−^ macrophages play critical roles in the regulation of inflammation and fibrosis (Figure 3) [29].

HFrEF can be understood as “cardiac weakness”, with myocardial infarction being its primary cause [30]. Large-scale necrosis of cardiomyocytes leads to a strong aseptic inflammatory response, further exacerbating systolic dysfunction [30]. Research shows that following myocardial injury, CCR2^+^ macrophages are mobilized through the CCL2/CCR2 signaling pathway and rapidly migrate to the injury site, where they secrete pro-inflammatory cytokines such as IL-1β, TNF-α, and IL-6 [31,32]. This triggers the activation of fibroblasts, promoting fibrosis and adverse cardiac remodeling [31,32]. The persistent infiltration and activation of CCR2^+^ macrophages result in excessive collagen deposition, ultimately causing further structural and functional damage to the heart [31,32].

In contrast, CCR2^−^ macrophages play a protective role during the early stages of HFrEF [33]. These macrophages proliferate locally and secrete anti-inflammatory factors, such as IGF-1, to suppress inflammation and promote cardiac tissue repair [15]. However, as cardiac injury progresses, the number of CCR2^−^ macrophages gradually decreases, leading to a reduction in their anti-fibrotic and reparative functions [17]. Throughout the pathological process of HFrEF, CCR2^+^ and CCR2^−^ macrophages are involved in bidirectional regulation of pro-inflammatory and anti-inflammatory responses, respectively [1].

HFpEF is often described as “cardiac stiffness”, and is closely associated with chronic conditions such as long-term hypertension and diabetes [34]. Its pathological hallmarks are impaired diastolic function and widespread cardiac fibrosis [34]. In HFpEF, systemic inflammation triggered by chronic diseases enhances the recruitment of CCR2^+^ macrophages, which further accelerates the progression of cardiac fibrosis [22,35]. CCR2^+^ macrophages promote fibrosis by secreting pro-fibrotic factors galectin-3, which activate cardiac fibroblasts, leading to excessive collagen deposition [22,35]. This results in myocardial stiffness and worsens diastolic dysfunction [22,35]. Additionally, the increased presence of CCR2^+^ macrophages is closely linked to a chronic inflammatory state, further driving the pathological progression of HFpEF [22].

Conversely, CCR2^−^ macrophages play anti-fibrotic and reparative roles in HFpEF [36]. These macrophages help maintain cardiac function by secreting anti-inflammatory factors and inhibiting the excessive activation of fibroblasts [36]. Through local proliferation, CCR2^−^ macrophages participate in the repair and remodeling of the heart, thereby slowing the progression of fibrosis and providing protection for cardiac function to some extent [36].

Despite recent advances in understanding the roles of macrophage subtypes in HF, several unresolved questions remain. For instance, how to precisely regulate the relative proportions and functions of CCR2^+^ and CCR2^−^ macrophages at different pathological stages to prevent excessive inflammation or fibrosis is a key focus of future research [3]. Additionally, while therapeutic strategies targeting the CCL2/CCR2 signaling pathway have shown some efficacy in animal models, their application in human HF patients requires further validation [37].

Future research should focus on several key areas. First, it is essential to investigate the dynamic changes and interaction mechanisms between CCR2^+^ and CCR2^−^ macrophages across different stages of HF. Second, the development of drugs that specifically target and regulate the functions of different macrophage subtypes may offer new avenues for personalized HF treatment. Finally, as anti-inflammatory and anti-fibrotic therapies continue to evolve, integrating macrophage modulation strategies with existing HF treatment options could significantly improve patient outcomes, opening new therapeutic horizons.

### 2.3. Atherosclerosis

Atherosclerosis (AS) serves as the key pathological basis for cardiovascular diseases, playing a pivotal role in their onset and progression [38,39]. Tissue-resident macrophages are particularly instrumental in driving AS development through processes such as foam cell formation and the regulation of plaque stability [38,39].

**Figure 4:**
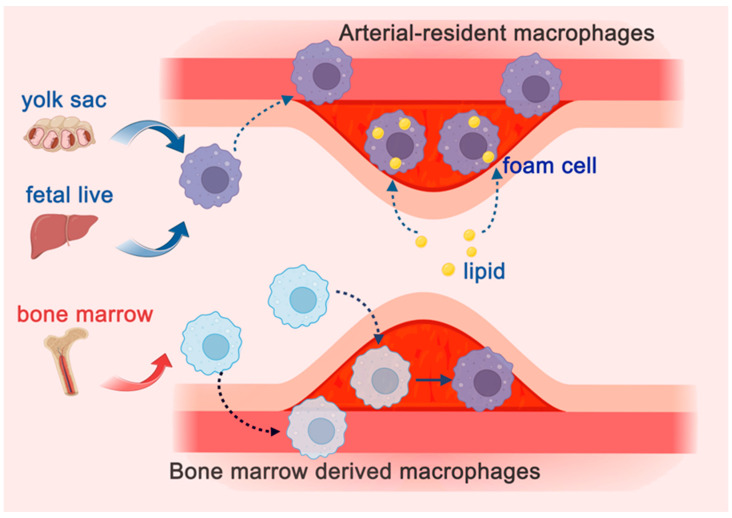
Origins and Role of Arterial-resident Macrophages in Atherosclerotic Plaque Formation and Progression. Macrophages originate from three sources: the primitive yolk sac, fetal liver monocytes, and bone marrow monocytes. Among them, macrophages derived from the first two sources are known as arterial-resident macrophages, which represent the predominant type of macrophage in the arterial intima under homeostatic conditions. Arterial-resident macrophages have the ability to engulf lipids and form the first foam cells within plaques, establishing the core of early plaque lesions. Additionally, they facilitate the chemotaxis of bone marrow monocytes to the subintimal space, thereby contributing to the initiation and growth of plaques. In the figure, dashed arrows indicate the movement trajectories of molecules or cells, while solid arrows represent transitions between distinct cell types.

Traditionally, macrophages in AS were thought to originate solely from bone marrow-derived monocytes [40,41]. However, recent findings from single-cell sequencing and genetic lineage tracing have revealed that macrophages have three distinct origins: the primitive yolk sac, fetal liver monocytes, and bone marrow monocytes [40,41]. Macrophages derived from the first two sources are collectively termed arterial-resident macrophages. Studies have shown that under steady-state conditions, the majority of macrophages in the arterial intima are arterial-resident macrophages (Figure 4) [42].

Using fate mapping and bone marrow chimera models, researchers have identified arterial-resident macrophages as key initiators of AS [42,43]. After ingesting lipids, these macrophages form the first foam cells within plaques, establishing the early lesion core and promoting the recruitment of bone marrow-derived monocytes to the subendothelial space, thereby jointly driving plaque growth [42,43]. Arterial-resident macrophages undergo extensive proliferation within plaques, and bone marrow monocytes can also partially convert into arterial-resident macrophages [44]. By the mid-to-late stages of AS, arterial-resident macrophages constitute up to 87% of macrophages in atherosclerotic lesions, underscoring their pivotal role in plaque development and progression [44]. Thus, arterial-resident macrophages are the predominant macrophages within plaques, playing a critical role in the initiation and progression of atherosclerosis (Figure 4).

**Figure 5:**
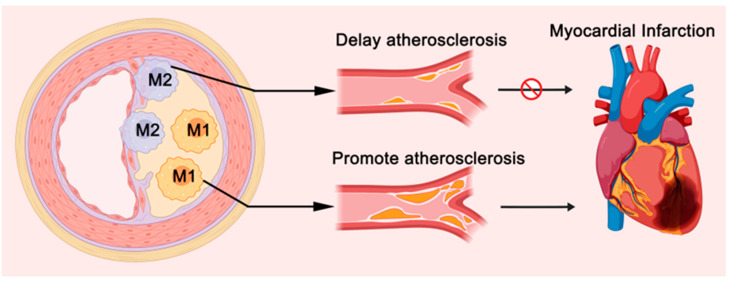
The Role of Different Subtypes of Arterial-resident Macrophages in Atherosclerotic Plaques and Cardiovascular Diseases. Arterial-resident macrophages exhibit heterogeneity and can be classified into pro-inflammatory M1 macrophages and anti-inflammatory M2 macrophages. In AS plaques, M1 macrophages are primarily located in unstable regions of the plaque, such as the shoulder and lipid core, areas prone to rupture, thereby accelerating the progression of AS and potentially leading to acute cardiovascular events, such as myocardial infarction. In contrast, M2 macrophages are mainly found in more stable regions, such as the fibrous cap of the plaque, where they help slow the progression of AS and reduce the incidence of acute cardiovascular diseases.

Similarly to macrophages derived from bone marrow monocytes, arterial-resident macrophages also exhibit heterogeneity, with subtypes classified as pro-inflammatory M1 macrophages and anti-inflammatory M2 macrophages (Figure 5) [45]. In atherosclerotic plaques, M1 macrophages are primarily located in regions prone to instability and rupture, such as the plaque shoulders and lipid cores [46,47]. These cells secrete pro-inflammatory cytokines such as TNF-α and IFN-γ, and also contribute to foam cell formation, accelerating the progression of atherosclerosis [46,47]. In contrast, M2 macrophages are mainly found in more stable regions of the plaque, such as the fibrous cap [46,47]. These cells produce anti-inflammatory cytokines such as TGF-β and IL-10, and promote collagen fiber secretion, which helps to stabilize the plaque and slow the progression of AS [46,47]. Research has demonstrated that the relative ratio of pro-inflammatory M1 to anti-inflammatory M2 macrophages is a critical factor in determining the initiation and progression of atherosclerotic plaques [46,47]. This balance between M1 and M2 arterial-resident macrophages plays a pivotal role in influencing plaque stability and the overall development of atherosclerosis.

Recent findings in the field of immunometabolism suggest that arterial-resident macrophages possess significant plasticity [48]. When key factors in the plaque microenvironment—such as oxygen levels, nutrient availability, and cytokine profiles—undergo changes, the metabolic pathways of arterial-resident macrophages can be reprogrammed, driving them to differentiate into either pro-inflammatory M1 or anti-inflammatory M2 macrophages [48]. This process, known as metabolic reprogramming, is central to the functional plasticity of arterial-resident macrophages [48].

**Figure 6:**
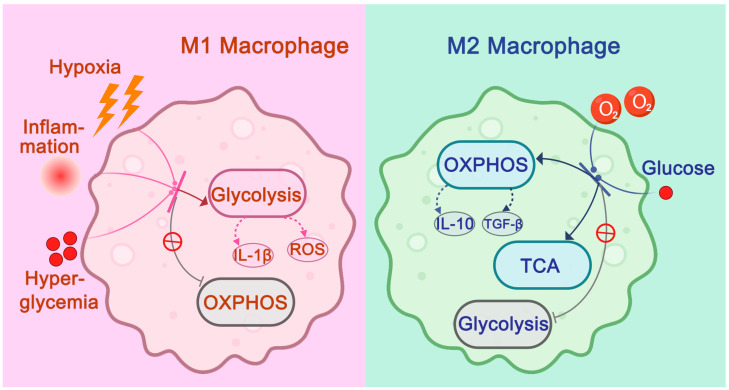
The Role of Glucose Metabolic Reprogramming in Regulating Arterial-resident Macrophage Differentiation into M1 and M2 Subtypes. In unstable regions of atherosclerotic plaques, like the plaque shoulder and necrotic core, factors such as hypoxia, hyperglycemia, and inflammatory signals increase glycolysis in arterial-resident macrophages while reducing mitochondrial oxidative phosphorylation (OXPHOS). This metabolic reprogramming drives more differentiation into pro-inflammatory M1 macrophages and less into anti-inflammatory M2 macrophages. Glycolysis also promotes the release of pro-inflammatory factors like IL-1β and ROS, worsening M1-induced tissue damage. In contrast, in more stable plaque areas, where oxygen supply is sufficient, glycolysis decreases, while OXPHOS and the TCA cycle are enhanced. This shift favors M2 differentiation, reducing M1 macrophages. M2 macrophages secrete anti-inflammatory mediators such as IL-10 and TGF-β, which help stabilize the plaque. In the figure, solid arrows depict activating or inhibitory effects of various factors within the plaque microenvironment on metabolic reprogramming pathways, while dashed arrows indicate pro-inflammatory or anti-inflammatory molecules derived from M1/M2 subtype cells through corresponding metabolic pathways.

Arterial-resident macrophages’ functionality in atherosclerotic plaques is heavily dependent on metabolic reprogramming [49]. Studies investigating the microenvironment of plaques have revealed that during plaque formation and progression, particularly in unstable regions such as the plaque shoulders and necrotic core, the limited diffusion distance of oxygen and increased cellular metabolic demands lead to localized hypoxia (Figure 6) [49,50,51,52,53,54,55,56,57]. Additionally, conditions such as hyperglycemia and the need for rapid energy production in response to inflammatory stimuli cause arterial-resident macrophages to shift from a resting state to a highly activated state. In such conditions, arterial-resident macrophages undergo a “Warburg effect”, a metabolic shift similar to that seen in cancer cells, where glycolysis is significantly upregulated for rapid energy production, while mitochondrial oxidative phosphorylation (OXPHOS) is reduced [49,50,51,52,53,54,55,56,57]. This metabolic shift drives arterial-resident macrophages more toward the M1 pro-inflammatory phenotype, while reducing differentiation into the M2 anti-inflammatory phenotype [49,50,51,52,53,54,55,56,57]. Intermediate products of glycolysis can also be used to synthesize various pro-inflammatory molecules, further exacerbating tissue damage mediated by M1 macrophages and contributing to plaque instability [54,57,58]. For example, the accumulation of succinate can promote the production of IL-1β and reactive oxygen species (ROS), amplifying M1-driven inflammatory responses and plaque destabilization [54,57,58]. In contrast, in more stable regions of the plaque, which are distant from the shoulder and lipid core, arterial-resident macrophages exhibit lower levels of glycolysis and higher levels of mitochondrial OXPHOS and the tricarboxylic acid (TCA) cycle [55,58]. This metabolic reprogramming shifts arterial-resident macrophages more toward the M2 phenotype, reducing M1 differentiation [55,58]. Compared to glycolysis, OXPHOS is a more efficient energy production pathway (32 ATP vs. 2 ATP per glucose molecule), and the sufficient energy produced supports the synthesis of anti-inflammatory mediators (e.g., IL-10 and TGF-β) and tissue repair molecules such as collagen fibers, thereby enhancing plaque stability [50,59,60,61,62]. This balance between glycolysis and OXPHOS in arterial-resident macrophages plays a crucial role in determining the inflammatory status and stability of atherosclerotic plaques, offering potential therapeutic targets for stabilizing plaques and preventing disease progression [50,59,60,61,62].

**Figure 7:**
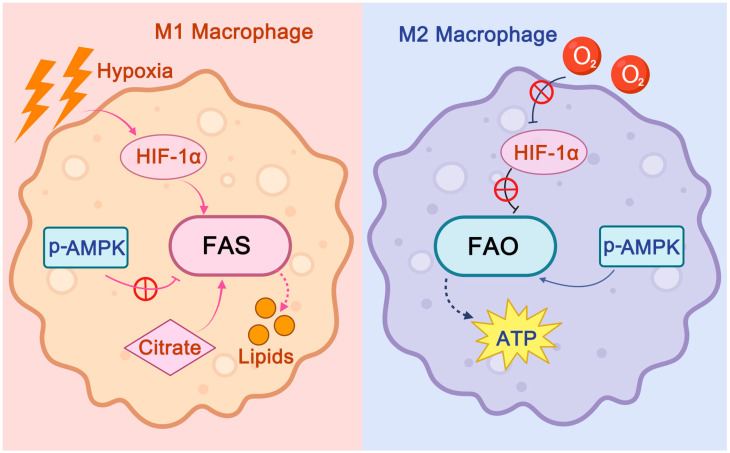
The Role of Fatty Acid Metabolic Reprogramming in Regulating Arterial-resident Macrophage Differentiation into M1 and M2 Subtypes. In unstable areas of the plaque, such as the shoulder and lipid core, hypoxia triggers the activation of HIF-1α. Additionally, metabolites like citrate within the plaque enhance FAS, which in turn promotes the differentiation of arterial-resident macrophages into the pro-inflammatory M1 subtype. The fatty acids produced through this process also encourage foam cell formation, further accelerating the progression of atherosclerosis. By contrast, in more stable regions of the plaque, such as the fibrous cap, where oxygen is sufficiently available, HIF-1α is suppressed. The relative energy scarcity in these regions activates AMPK, stimulating FAO. This process provides a crucial energy source for macrophages and supports their differentiation into the anti-inflammatory M2 subtype. In the figure, solid arrows depict activating or inhibitory effects on fatty acid metabolic pathways or key molecules involved, while dashed arrows indicate resultant metabolites.

Additionally, fatty acid metabolism plays a crucial regulatory role in macrophage polarization and function (Figure 7) [49,63,64]. In unstable regions of atherosclerotic plaques, such as the plaque shoulders and lipid core, hypoxia-inducible factor HIF-1α is activated by the low oxygen environment [49,63,64]. Both hypoxia and HIF-1α can upregulate key enzymes involved in fatty acid synthesis (FAS), while the anaerobic glycolysis and TCA cycle within the plaque provide sufficient precursors, such as citrate, for FAS [49,63,64]. This results in enhanced FAS, which drives arterial-resident macrophages toward the M1 pro-inflammatory macrophage phenotype [49,63,64]. The fatty acids synthesized through FAS are critical for M1 macrophage functions, including membrane remodeling (new membrane formation is a central process in M1 activation) and the production of inflammatory mediators like TNF-α and IFN-γ [62,64,65]. Furthermore, fatty acids generated by FAS contribute to foam cell formation, thereby exacerbating the progression of atherosclerosis [64]. In contrast, in more stable regions of the plaque, such as the fibrous cap, oxygen supply is relatively sufficient, leading to inhibition of HIF-1α [7,61,63,66]. At the same time, the complex inflammatory repair responses in the plaque consume significant energy, resulting in relative energy scarcity, which activates AMP-activated protein kinase (AMPK) [7,61,63,66]. This activation promotes fatty acid oxidation (FAO) in arterial-resident macrophages [7,61,63,66]. FAO is a major energy source for macrophages, with a single fatty acid molecule (e.g., palmitate) producing over 129 ATP molecules [67]. Increased FAO drives the differentiation of macrophages toward the M2 anti-inflammatory phenotype (some researchers propose that M2 activation is entirely dependent on FAO) and supports the production of anti-inflammatory mediators such as TGF-β and IL-10 [49,64,68]. FAO also provides sufficient energy for phagocytic activity and the inflammation resolution process in M2 macrophages [49,64,68]. Additionally, FAO is often accompanied by triglyceride lipolysis, which reduces lipid droplet accumulation within M2 macrophages and inhibits foam cell formation [69]. This metabolic shift toward FAO in stable regions helps maintain plaque stability by promoting M2 polarization and reducing the formation of foam cells, ultimately contributing to the resolution of inflammation and the stabilization of atherosclerotic plaques [69].

The complex pathological mechanisms of atherosclerosis and the metabolic reprogramming of arterial-resident macrophages offer promising avenues for further research. Targeting macrophage metabolic reprogramming opens new strategies for the treatment of cardiovascular diseases. We envision that inhibiting glycolysis and FAS pathways in M1 macrophages, or enhancing FAO in M2 macrophages, may effectively reduce foam cell formation, mitigate inflammatory responses, and slow the progression of atherosclerosis. Future research aimed at further elucidating the metabolic reprogramming mechanisms of arterial-resident macrophages in AS holds the potential to develop highly specific therapeutic targets. These targets could not only improve plaque stability and reduce inflammation but also significantly enhance clinical outcomes. This approach offers a novel therapeutic window for the treatment of atherosclerosis and provides a solid theoretical and practical foundation for the prevention and treatment of other cardiovascular diseases.

### 2.4. Hypertension and Pulmonary Arterial Hypertension

In angiotensin II-induced hypertension models, the expansion of macrophages occurs mainly through two mechanisms: first, through the CCR2 pathway, which mediates the recruitment of circulating monocytes into local tissues where they differentiate into macrophages; second, through the self-proliferation of tissue-resident macrophages in the local microenvironment [8,36]. These macrophages expand significantly in the heart, kidneys, and perivascular adipose tissue, driving local inflammatory responses and contributing to the pathological progression of hypertension [70,71,72,73]. Notably, while the role of tissue-resident macrophages in maintaining homeostasis is widely recognized, it remains challenging to distinguish their specific contributions from those of monocyte-derived macrophages in hypertension [36]. Both populations are involved in initiating and sustaining hypertension-associated inflammation [36]. Macrophages exacerbate local inflammation by secreting pro-inflammatory factors such as IL-1β, TNF-α, and reactive oxygen species (ROS), which drive vascular remodeling processes, including smooth muscle cell proliferation, vascular wall thickening, and endothelial dysfunction [74,75]. These processes ultimately lead to impaired sodium excretion and kidney damage [74,75]. Additionally, the phenotypic plasticity of macrophages during the progression of hypertension is critical [71,74,76,77]. M1 macrophages promote early blood pressure elevation through pro-inflammatory mechanisms, while, in the later stages of the disease, macrophages may transition to an M2 phenotype, which possesses potential anti-inflammatory and tissue repair functions [71,74,76,77]. Based on these findings, therapeutic strategies targeting macrophages, such as inhibiting the CCR2 pathway to reduce macrophage recruitment, may help alleviate vascular remodeling and the pathological responses associated with hypertension [36,71].

In the development of pulmonary arterial hypertension (PH), tissue-resident macrophages play a pivotal role by regulating the recruitment of circulating monocytes through the CX3CR1 and CCR2 chemokine pathways [78]. This significantly enhances pulmonary inflammation and is critical in driving pulmonary vascular remodeling [78]. Tissue-resident macrophages promote the proliferation of pulmonary vascular smooth muscle cells, endothelial cell damage, and fibrosis through the secretion of pro-inflammatory cytokines such as IL-1β, IL-6, and TNF-α, which ultimately lead to the thickening and stiffening of the pulmonary vessel walls [78]. The phenotypic plasticity of tissue-resident macrophages is also essential in PH [78]. M1 macrophages promote pulmonary vascular lesions through pro-inflammatory mechanisms, while M2 macrophages may mediate tissue repair in later stages through anti-inflammatory pathways [78,79]. Inhibition of the CX3CR1/CX3CL1 pathway can reduce macrophage accumulation in the lungs, significantly alleviating pulmonary vascular remodeling, thereby offering a new potential therapeutic target for PH [78,79]. A deeper understanding of these mechanisms not only sheds light on the pathophysiology of PH but also provides theoretical support for the development of future precision therapy strategies.

## 3. Conclusions and Future Perspectives

The functional heterogeneity and bidirectional regulatory mechanisms of TRMs in cardiovascular diseases have established a new paradigm for therapeutic development [80]. Current research reveals that CCR2^+^ and CCR2^−^ subsets exhibit functional divergence in pro-inflammatory/fibrotic vs. anti-inflammatory/tissue repair processes [14,31], with metabolic reprogramming of M1 and M2 subpopulations emerging as novel intervention targets [4]. However, this field faces three critical challenges: (1) The traditional M1/M2 classification inadequately reflects in vivo complexity [14], while surface markers like CCR2 lack pathological specificity [31]; (2) Shared glycolytic/oxidative phosphorylation metabolic nodes between macrophages and cardiomyocytes create dual risks of disrupting immune regulation and myocardial metabolism during targeted interventions [81], potentially triggering lethal arrhythmias; (3) Conventional nanocarriers struggle to overcome biological delivery barriers [82]. These bottlenecks have spurred innovations such as “metabolic fine-tuning” strategies [81] and intelligent delivery systems like pH-sensitive liposomes [82].

Future advancements will hinge on multi-omics technologies and novel model systems: Spatially resolved transcriptomics can map macrophage functional landscapes in peri-infarct zones [83], while humanized organ-on-chip platforms integrating hemodynamics and comorbidities (e.g., diabetes) will address species-specific disparities (80% resident macrophages in murine hearts vs. 40% in humans) [84] and limitations of compressed pathological timelines [85]. Combinatorial therapies (e.g., CCR2 inhibitors with PD-1 blockers) [86] and CRISPR-mediated spatiotemporally controlled epigenetic regulation [87] demonstrate synergistic potential. The clinical application of real-time monitoring technologies, such as CD206-targeted PET tracers [88], heralds the dawn of personalized cardiovascular immunotherapy.

Current research is driving a paradigm shift from broad-spectrum anti-inflammation to precision immune remodeling [14]. Through integration of single-cell omics, gene editing, and smart biomaterials [89], precision modulation of cardiac macrophages is poised to overcome therapeutic impasses within the next decade [14], redefining cardiovascular disease management. This transformation demands not only continuous fundamental innovation but also the establishment of cross-scale translational research frameworks to bridge mechanistic discoveries to clinical benefits, ultimately achieving a closed loop from bench to bedside [80].

## Abbreviations

Abbreviation	Full Name
TRMs	Tissue-Resident Macrophages
CRMs	Cardiac Resident Macrophages
CCR2	C-C Chemokine Receptor Type 2
IL-1β	Interleukin-1β
TNF-α	Tumor Necrosis Factor-alpha
IL-10	Interleukin-10
TGF-β	Transforming Growth Factor-beta
PDGF-C	Platelet Derived Growth Factor C
MI	Myocardial Infarction
HF	Heart Failure
HFrEF	Heart Failure with Reduced Ejection Fraction
HFpEF	Heart Failure with Preserved Ejection Fraction
AS	Atherosclerosis
IGF-1	Insulin-like growth factor 1
OXPHOS	Oxidative Phosphorylation
TCA	Tricarboxylic Acid Cycle
FAS	Fatty Acid Synthesis
FAO	Fatty Acid Oxidation
HIF-1α	Hypoxia-Inducible Factor-1α
AMPK	AMP-Activated Protein Kinase
ROS	Reactive Oxygen Species
CCL2	C-C Motif Chemokine Ligand 2
CX3CR1	C-X3-C Motif Chemokine Receptor 1
LYVE1	Lymphatic Vessel Endothelial Hyaluronan Receptor 1
MHC-II	Major Histocompatibility Complex Class II
PH	Pulmonary arterial hypertension
